# Growth and fermentation of D-xylose by *Saccharomyces cerevisiae* expressing a novel D-xylose isomerase originating from the bacterium *Prevotella ruminicola* TC2-24

**DOI:** 10.1186/1754-6834-6-84

**Published:** 2013-05-30

**Authors:** Ronald E Hector, Bruce S Dien, Michael A Cotta, Jeffrey A Mertens

**Affiliations:** 1 Bioenergy Research Unit, United States Department of Agriculture, Agricultural Research Service, National Center for Agricultural Utilization Research, Peoria, IL 61604, USA

**Keywords:** *Saccharomyces cerevisiae*, *Prevotella ruminicola*, *Bacteroides*, D-xylose isomerase, Ethanol, Lignocellulose

## Abstract

**Background:**

*Saccharomyces cerevisiae* strains expressing D-xylose isomerase (XI) produce some of the highest reported ethanol yields from D-xylose. Unfortunately, most bacterial XIs that have been expressed in *S*. *cerevisiae* are either not functional, require additional strain modification, or have low affinity for D-xylose. This study analyzed several XIs from rumen and intestinal microorganisms to identify enzymes with improved properties for engineering *S*. *cerevisiae* for D-xylose fermentation.

**Results:**

Four XIs originating from rumen and intestinal bacteria were isolated and expressed in a *S*. *cerevisiae* CEN.PK2-1C parental strain primed for D-xylose metabolism by over expression of its native D-xylulokinase. Three of the XIs were functional in *S*. *cerevisiae*, based on the strain’s ability to grow in D-xylose medium. The most promising strain, expressing the XI mined from *Prevotella ruminicola* TC2-24, was further adapted for aerobic and fermentative growth by serial transfers of D-xylose cultures under aerobic, and followed by microaerobic conditions. The evolved strain had a specific growth rate of 0.23 h^-1^ on D-xylose medium, which is comparable to the best reported results for analogous *S*. *cerevisiae* strains including those expressing the *Piromyces* sp. E2 XI. When used to ferment D-xylose, the adapted strain produced 13.6 g/L ethanol in 91 h with a metabolic yield of 83% of theoretical. From analysis of the *P*. *ruminicola* XI, it was determined the enzyme possessed a *V*_*max*_ of 0.81 μmole/min/mg protein and a *K*_*m*_ of 34 mM.

**Conclusion:**

This study identifies a new xylose isomerase from the rumen bacterium *Prevotella ruminicola* TC2-24 that has one of the highest affinities and specific activities compared to other bacterial and fungal D-xylose isomerases expressed in yeast. When expressed in *S*. *cerevisiae* and used to ferment D-xylose, very high ethanol yield was obtained. This new XI should be a promising resource for constructing other D-xylose fermenting strains, including industrial yeast genetic backgrounds.

## Background

Concern regarding the risks in climate change associated with greenhouse gas emissions is driving policies promoting lower CO_2_ emissions. In the United States, one third of CO_2_ emissions are from transportation despite the blending of 14 billion gallons of ethanol originating from grains (e.g. corn) (http://www.ethanolrfa.org accessed Feb. 2013). It is expected that further growth in biofuel production will need to rely on lignocellulosic feedstocks. Lignocellulose includes agricultural and forest wastes as well as dedicated energy crops, such as perennial grasses or tree plantations. Besides being available in larger quantities than grains, these feedstocks do not impinge on the food and feed market, are more CO_2_ neutral, and will not interfere with current ethanol production.

Several technologies are being developed that use lignocellulose for producing biofuels. The biochemical route is one of the most advanced in terms of development. In this route, carbohydrates are extracted, usually in the form of monosaccharides, and fermented to ethanol. While several efforts are underway at the production scale, there is a continued need for further strain development.

Industrial ethanol is produced using *Saccharomyces* yeast. *Saccharomyces* yeasts are favored because of their excellent yield, tolerance of low pH that discourages the growth of spoilage microbes, ability to grow aerobically for efficient cell generation, and robustness. However, plant cell walls contain a mixture of sugars, including D-xylose, which *Saccharomyces* spp. cannot consume. Over two decades of effort have been expended on developing *S*. *cerevisiae* stains that ferment D-xylose and (more recently) L-arabinose and research continues in enhancing the productivity of D-xylose fermentation [1,2].

Current research efforts are focused on improving D-xylose transport into the cell, conversion of D-xylose to D-xylulose, and optimization of the non-oxidative pentose phosphate pathway that feeds into glycolysis [3,4]. Rational strategies have been supplemented by evolutionary adaptation using continuous and serial batch cultures [5-7]. This study focuses on the second area.

Two strategies have been pursued for converting D-xylose to D-xylulose. The earliest effort consisted of expressing two genes from the native D-xylose-fermenting yeast *Scheffersomyces stipitis*[8,9]. *S*. *stipitis* converts D-xylose to xylitol and xylitol to D-xylulose by the actions of D-xylose reductase and xylitol dehydrogenase. Simple expression of the genes in *S*. *cerevisiae* favors production of xylitol over ethanol because the preference of the reductase for NADPH and of the dehydrogenase for NAD^+^. Additionally, conversion of D-xylulose to D-xylulose-5-phosphate is rate limiting [10]. Redox engineering and fine-tuning D-xylulokinase activity have been successful in reducing xylitol production.

Most bacteria transform D-xylose to D-xylulose in a single step that relies on the enzyme D-xylose isomerase (XI). Efforts to express bacterial D-xylose isomerases in yeast have been largely unsuccessful. A breakthrough occurred when a D-xylose isomerase was discovered in an anaerobic fungus and this D-xylose isomerase was successfully expressed in *S*. *cerevisiae*[11,12]. While initial growth rates were slow, over expressing genes related to D-xylose fermentation and evolutionary adaptation considerably improved its performance. Still this approach is not without its disadvantages stemming from problems related to expression of D-xylose isomerase and the enzyme’s poor kinetic properties. Until recently, the D-xylose isomerase from the anaerobic fungus *Piromyces* sp. E2 was the only XI gene that functioned in *S*. *cerevisiae*. However, due to its low affinity for D-xylose (*K*_*m*_ from 20 to 90 mM) [12-14], the search for new D-xylose isomerases that function in *S*. *cerevisiae* has continued.

Many of these new XIs do not confer the ability to grow on D-xylose without first modifying the D-xylose isomerase or adapting the host strain. For example, expression of the *Clostridium phytofermentans* XI in *S*. *cerevisiae* was shown in two separate studies to require codon-optimization and strain adaptation [14,15]. The *Ruminococcus flavefaciens* XI was recently expressed in *S*. *cerevisiae*. Yeast strains expressing several versions of this XI failed to grow aerobically in D-xylose medium, despite one of the expressed XI enzymes having a high specific activity [16]. The XI gene from an anaerobic rumen fungus, *Orpinomyces*, was also expressed in *S*. *cerevisiae* and produced a high specific activity (1.73 U/mg lysate). D-xylose consumption was limited to 10 g/L in 140 hours [17]. However, further strain modification by addition of the sugar transporter *SUT1* resulted in 15 g/L D-xylose consumed over the same time period.

The goal of this study was to identify novel D-xylose isomerases that function when expressed in *S*. *cerevisiae*. Rumen and intestinal bacteria were used as the source of these additional XIs. The rumen and intestinal microbial ecosystems are promising niches to mine for new XIs due to the prevalence of xylan degrading microorganisms in these environments. A D-xylose isomerase from the rumen bacterium *Prevotella ruminicola* TC2-24 was discovered that conferred the ability to grow on D-xylose medium when expressed in *S*. *cerevisiae* without strain adaption. The strain was further improved by adaptation under aerobic and fermentative conditions. The evolved strain was compared to an adapted *S*. *cerevisiae* strain expressing the D-xylose reductase (XR) and xylitol dehydrogenase (XD) genes from *Scheffersomyces stipitis*.

## Results and discussion

### Cloning and expression of bacterial D-xylose isomerases in *Saccharomyces cerevisiae*

*Prevotella* spp. and *Bacteroides* spp. are among the most common xylan-degrading microorganisms isolated from the bovine rumen and human colon, representing a dominant phylum (i.e. *Bacteroidetes*) in these ecosystems. Bacterial D-xylose isomerases have been reported with much higher affinity for D-xylose when compared to the fungal *Piromyces* sp. E2 or *Orpinomyces* D-xylose isomerases. With the goal of identifying D-xylose isomerases with increased affinity for D-xylose, D-xylose isomerase genes from *Bacteroides* and *Prevotella* spp. were isolated and expressed in *S*. *cerevisiae*. D-xylose isomerase genes were isolated from three *Bacteroides* spp. (*B*. *uniformis*, *B*. *distasonis*, and *B*. *ovatus*) and *P*. *ruminicola* strain TC2-24. Attempts to isolate D-xylose isomerase genes from other *P*. *ruminicola* strains failed (see methods). Several *Prevotella* spp., including the sequenced type strain *P*. *ruminicola* 23, have been reported to be missing the gene for D-xylose isomerase. When grown on D-xylose, these strains have detectable but low D-xylose isomerase activity and high D-xylulokinase activity [18,19]. Yet, they lack an obvious D-xylose reductase and xylitol dehydrogenase pathway. It is unclear whether the absence of a D-xylose isomerase gene is due to an incomplete genome sequence or if they possess an alternate mechanism to convert D-xylose to D-xylulose. *P*. *ruminicola* strain TC2-24 shows many similarities with *P*. *ruminicola* 23 but does exhibit differences when compared to this type strain [20,21]. Although we have not investigated further, the presence of a D-xylose isomerase in TC2-24 may be another characteristic of this group of “*P*. *ruminicola* 23-like” strains. The D-xylose isomerase identified from *P*. *ruminicola* TC2-24 was 439 amino acids long and 79% to 85% identical to D-xylose isomerases from other *Prevotella* spp. The TC2-24 XI was 79% identical to the *Piromyces* sp. E2 and *Orpinomyces* XIs and only 53% identical to the *C*. *phytofermentans* XI. The *Bacterodies* spp. XIs analyzed in this study were 81% to 83% identical to the fungal isomerases.

### Aerobic growth in D-xylose medium

**Figure 1:**
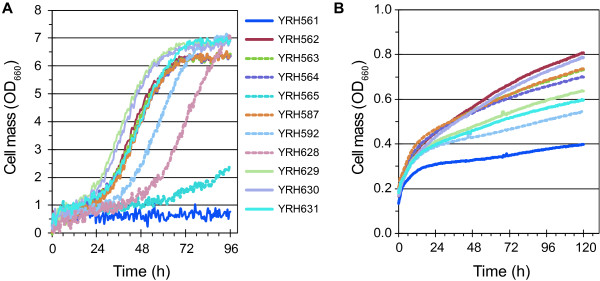
**Comparison of *Saccharomyces cerevisiae *strains engineered to express various D-xylose isomerase and D-xylulokinase genes. A**) Strains were cultured under aerobic conditions using YP medium with 50 g/L D-xylose. Cultures were incubated at 30°C, shaking at 1000 rpm using a BioLector®. Cell density was measured every 30 minutes. Data shown are mean values from experiments performed in triplicate. **B**) Strains were cultured under microaerobic conditions using YP medium with 50 g/L D-xylose. Cultures were incubated at 30°C using a Bioscreen C™. Cell density was measured every 30 minutes. Data shown are the average values from three biological replicates. The standard deviation for most values was less than 5%. Panel **B** uses the same legend as in panel **A**.

To determine if the D-xylose isomerase genes were functional, each gene was expressed in *S*. *cerevisiae* along with the *S*. *cerevisiae* D-xylulokinase gene, *XKS1*, and screened for growth on D-xylose in aerobic liquid culture (Figure 1A). The control strain YRH561, which did not express D-xylose metabolism genes, did not grow. Strain YRH562, expressing the *Piromyces* sp. E2 XI (and *S*. *cerevisiae XKS1*), was included for comparison. YRH562 had a specific growth rate of 0.07 h^-1^ on D-xylose medium under aerobic growth conditions (Table 1). Several of the bacterial isomerases conveyed the ability for growth on D-xylose at specific growth rates similar to YRH562. As expected, specific activity of a D-xylose isomerase in the *S*. *cerevisiae* strain was correlated with the growth of that strain on D-xylose (data not shown). For example, the *Bacteroides ovatus* XI expressed in strain YRH565 had very low activity and this strain also grew poorly in D-xylose medium (Figure 1A). Equilibrium kinetics of the D-xylose isomerase reaction do not favor production of D-xylulose and co-expression with a D-xylulokinase has been recommend by prior studies to help pull D-xylose into the pathway. As evidence, strains not over expressing D-xylulokinase (YRH628 in Figure 1A) grew slower compared to strains expressing elevated levels of D-xylulokinase. Endogenous XK expression in YRH628 allowed for some growth on D-xylose.

**Table 1:** Specific growth rates for aerobic growth in D-xylose medium

**Strain**	**D-xylose isomerase**	**D-xylulokinase**	**Specific growth rate, μ(h**^**-1**^ ± **SD)**
YRH561	none	none	n.d.^a^
YRH562	*Piromyces* sp. E2	*S. cerevisiae*	0.07 ± 0.002
YRH563	*B. uniformis*	*S. cerevisiae*	0.07 ± 0.007
YRH564	*B. distasonis*	*S. cerevisiae*	0.06 ± 0.004
YRH565	*B. ovatus*	*S. cerevisiae*	n.d.
YRH587	*P*. *ruminicola*	*S*. *cerevisiae*	0.08 ± 0.005
YRH592	*B*. *uniformis*	*B*. *uniformis*	0.07 ± 0.012
YRH628	*P*. *ruminicola*^opt^	none	0.06 ± 0.004
YRH629	*P*. *ruminicola*^opt^	*S*. *cerevisiae*	0.07 ± 0.005
YRH630	*Piromyces* sp. E2	*P*. *ruminicola*^opt^	0.07 ± 0.006
YRH631	*P*. *ruminicola*^opt^	*P*. *ruminicola*^opt^	0.06 ± 0.005
YRH1114	*P*. *ruminicola*^opt^	*P*. *ruminicola*^opt^	0.23 ± 0.024

^Opt^ Codon-adapted for expression in *S. cerevisiae.*

^a^ n.d.: Growth in D-xylose medium was poor or not detected.

Strain YRH587, expressing the XI from *Prevotella ruminicola* (TC2-24) and *ScXKS1* grew as well as strain YRH562 expressing the *Piromyces* XI and *ScXKS1*. Specific growth rates for the two strains were also comparable (Table 1) indicating that the *P*. *ruminicola* XI was functional when expressed in *S*. *cerevisiae*. Codon optimization of the *P*. *ruminicola* XI (YRH629) did not enhance growth relative to YRH587. Additionally, the *P*. *ruminicola* D-xylulokinase was substituted for the *ScXKS1* gene. YRH631 cells expressing the bacterial XK gene had a similar growth rate as the YRH629 strain expressing the *S*. *cerevisiae* XK, indicating that the *P*. *ruminicola* XK was also functionally expressed. Strain YRH630, expressing the *Piromyces* XI gene and the *P*. *ruminicola* XK (Figure 1A), grew as well as strain YRH562 expressing the *Piromyces* XI gene with the *S*. *cerevisiae* XK. Cells expressing the *B*. *uniformis* XK with the *B*. *uniformis* XK reached a higher cell density with the *B*. *uniformis* XK compared to the strain expressing the *S*. *cerevisiae* XK (YRH592 vs. YRH563). These results demonstrate that the *B*. *uniformis* and *P*. *ruminicola* XKs are active in *S*. *cerevisiae*.

Next, the strains were evaluated for growth under oxygen-limited conditions using the Bioscreen C™, a microtiter based system for measuring microbial growth rates. Aerobic growth studies were performed using a 6-edged flower shaped microtiter plate in the BioLector® under conditions that were optimized for high oxygen transfer rate [22]. Round microtiter plates such as those used in the Bioscreen C™ have been shown to provide lower oxygen transfer rates [22], and decreased oxygen levels are a likely cause of the poor cell growth seen when the Bioscreen C™ is used to culture cells on respiratory carbon sources like ethanol [23]. All of the strains grew poorly when cultured using the Bioscreen C™. The best strains reached a cell density of less than 0.8 OD_660_ in 96 hours (Figure 1B). Such poor growth under these conditions suggested that adaptation to an oxygen-limited environment would be beneficial.

### Adaptation for improved D-xylose fermentation

**Figure 2:**
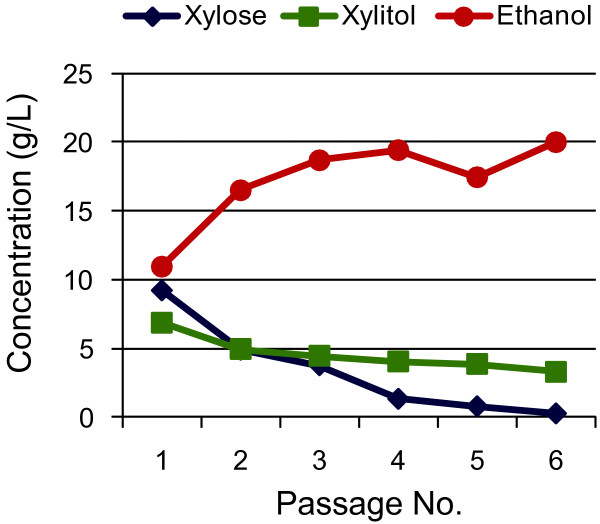
**Adaptation of strain YRH631 to create strain YRH1114.** Strain YRH631, expressing the *Prevotella ruminicola* XI and XK genes was cultured under microaerobic conditions and passaged every seven days. Remaining D-xylose and the fermentation products ethanol and xylitol was measured prior to each passage. Data shown are from one of two replicates.

Adaptation, either by serial batch or continuous cell culture, has been successful for improving growth and fermentation of *S*. *cerevisiae* strains expressing different D-xylose isomerase genes [6,14] as well as for cells expressing the reductase/dehydrogenase genes for D-xylose metabolism [7]. We used serial passage of cells to select for spontaneous changes that resulted in increased growth under fermentative conditions (microaerobic). YRH631 was grown in YPX and transferred weekly for a total of six transfers. Residual D-xylose and the fermentation products ethanol and xylitol were measured for each culture (Figure 2). Steady strain improvement was observed as greater D-xylose utilization and increased production of ethanol (Figure 2). Acetate (not shown) remained constant at 2 g/L.

**Figure 3:**
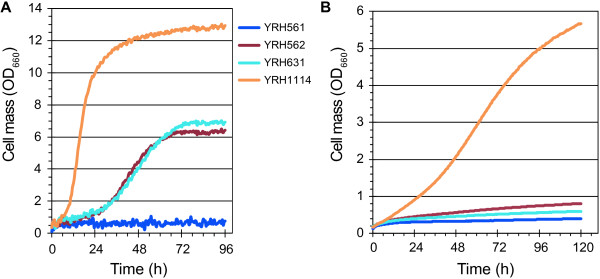
**Growth curves for the adapted *Saccharomyces cerevisiae *strain engineered to express the *P. ruminicola *D-xylose isomerase and D-xylulokinase genes. A**) Strains were cultured under aerobic conditions in YP medium with 50 g/L D-xylose. Cultures were incubated at 30°C, shaking at 1000 rpm using a BioLector®. Cell density was measured every 30 minutes. Data shown are mean values from experiments performed in triplicate. **B**) Strains were cultured under microaerobic conditions using YP medium with 50 g/L D-xylose. Cultures were incubated at 30°C using a Bioscreen C™. Cell density was measured every 30 minutes. Data shown are the average values from three biological replicates. The standard deviation for most values was less than 5%. Panel **B** uses the same legend as in panel **A**.

The adapted strain YRH1114 was isolated from the last serial culture and subsequently evaluated for growth in D-xylose medium under aerobic and microaerobic conditions (Figure 3A, B and Table 1). Strains expressing the *Piromyces* XI (YRH562) and the unadapted strain expressing the *P*. *ruminicola* XI (YRH631) were included for comparison. The adapted strain, YRH1114, showed a significant increase in growth under both culture conditions compare to the unadapted strain. The specific growth rate for YRH1114 was 0.23 h^-1^, an increase of 3.8-fold compared to the unadapted strain, is among the highest reported growth rates which range from 0.01 h^-1^ to 0.22 h^-1^ for a *S*. *cerevisiae* strain expressing D-xylose isomerase [6,13,14,16,17,24-26].

### D-xylose fermentation

**Figure 4:**
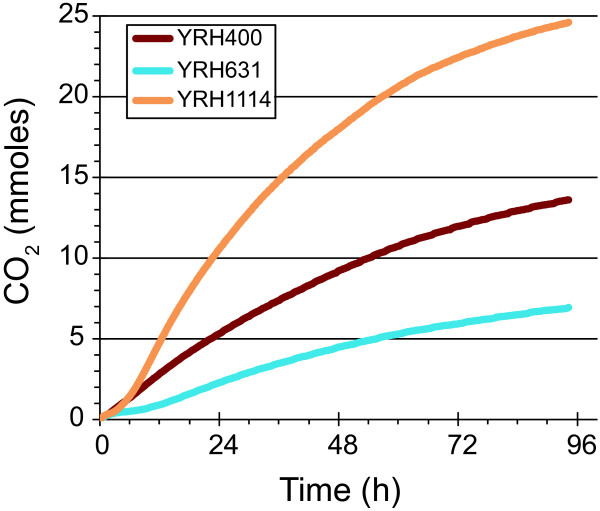
**Comparison of D-xylose fermentation using *Saccharomyces cerevisiae *strains engineered to express the *P. ruminicola *D-xylose isomerase and D-xylulokinase genes vs. expression of the *Scheffersomyces stipitis *D-xylose reductase and xylitol dehydrogenase genes.** Fermentations were performed using YP medium with 50 g/L D-xylose. Pressure was measured every 15 minutes and converted to mmoles of CO_2_. Data shown are from a single representative fermentation from experiments performed in triplicate.

The adapted XI yeast strain (YRH1114) was also evaluated for its ability to ferment D-xylose to ethanol using microaerobic conditions. For comparison, the unadapted yeast strain expressing the *P*. *ruminicola* XI and XK was also included in the experiment. Finally, YRH400, a D-xylose fermenting yeast that expresses the alternate D-xylose utilization genes (*S*. *stipitis* XR and XD, with elevated Sc*XKS1*) in a commercial yeast background was also included; this strain is currently our best performing strain for D-xylose fermentation [27]. Progress of the fermentations was monitored by measuring production of CO_2_ (Figure 4): one mole of CO_2_ is produced for each mole of ethanol. YRH1114 outperformed these strains producing 13.6 g/L ethanol with a metabolic yield of 82.9% of theoretical (Table 2). The unadapated XI yeast strain YRH631 had a much lower ethanol titer (4.1 g/L) even though the metabolic yield was closer to that of the adapted strain YRH1114. The difference in final ethanol concentration between the adapted and unadapted strain arose primarily from differences in D-xylose consumption rate and specific ethanol productivity. The adapted strain had a significantly higher ethanol productivity compared to the unadapted strain also expressing the *P*. *ruminicola* XI.

**Table 2:** Fermentation products

**Strain**	**D-xylose consumed [g/L]**	**Xylitol [g/L]**	**Glycerol [g/L]**	**Ethanol [g/L]**	**Sp. Ethanol productivity [g/g**_**CDW**_**/h]**	**Ethanol yield [% Theoretical]**	**Carbon recovery [%]**
YRH400	27.9 ± 1.00	14.3 ± 1.79	0.5 ± 0.04	6.9 ± 0.13	0.028 ± 0.0013	48.3 ± 1.07	95.5 ± 0.06
YRH631	11.7 ± 0.52	3.0 ± 0.08	0.8 ± 0.06	4.1 ± 0.21	0.015 ± 0.0002	68.6 ± 0.99	100.0 ± 0.01
YRH1114	32.1 ± 0.45	4.8 ± 0.13	1.8 ± 0.13	13.6 ± 0.08	0.041 ± 0.0015	82.9 ± 1.66	91.1 ± 0.02

Data shown represent the mean from triplicate experiments ± SD.

The most notable comparison is between the adapted YRH1114 strain and YRH400. Even though YRH400 had likewise been adapted and possessed an industrial yeast background, YRH1114 produced nearly twice as much ethanol (Table 2). This difference is also apparent in lower specific ethanol productivity for YRH400 compared to YRH1114. While YRH400 consumed nearly as much D-xylose, it funneled approximately 50% of the D-xylose to the production of xylitol, which is approximately three times as much as the XI expressing strains. This difference in xylitol production is reflected in the much lower metabolic ethanol yield for YRH400 compared to the other strains. Most strains using the XR/XD pathway suffer from low ethanol yield, although an adapted strain using this pathway was recently reported to have a 79% of theoretical ethanol yield from D-xylose [28].

Selective ethanol production is a hallmark of XI expressing strains because the isomerase step avoids the redox imbalance observed for expression of the genes from *S*. *stipitis*. This redox imbalance arises because each enzyme (see introduction) favors an alternative electron carrier. This imbalance has been partially relieved by redox engineering of the XR and XD enzymes [29-32]. Increases in ethanol yield during D-xylose fermentation are seen for some redox engineered strains, but ethanol yields from D-xylose for these strains are still 10% to 19% lower than the yield obtained with the adapted strain described in this study. Thus, the adapted strain expressing the *P*. *ruminicola* XI and XK compares favorably to results reported for other strains when cultured on D-xylose. It is also likely the performance of the *P*. *ruminicola* XI can be further improved by expressing the gene in a more robust yeast strain such as D5A, which was used for constructing YRH400 [27].

### Kinetic parameters

As discussed previously, one concern with XI-based systems is poor affinity of the enzyme for D-xylose. *S*. *cerevisiae* does not possess a native D-xylose transporter and intracellular D-xylose concentrations might be rate limiting. Therefore, it was of interest to measure the Michaelis-Menten kinetic constants for the *P*. *ruminicola* XI in comparison to other D-xylose isomerases. Enzyme kinetic parameters for the *P*. *ruminicola* XI were measured for the pre and post adapted strains.

The *K*_*m*_ for the *P*. *ruminicola* XI was 40 mM and 34 mM before and after adaptation, respectively. While the *K*_*m*_ measurement using lysate from the adapted strain was slightly lower, this difference was not statistically significant. The *K*_*m*_ for the *P*. *ruminicola* XI appears to be more favorable than the *K*_*m*_ for *Piromyces* sp. E2 XI, which was observed to be 51 mM in this study and varies considerably in the literature (Table 3). Thus, compared to bacterial XIs expressed in yeast, and to most reported *K*_*m*_ values for the *Piromyces* XI, the *P*. *ruminicola* XI has a higher affinity for D-xylose.

**Table 3:** **Kinetic properties of the *Prevotella ruminicola *D-xylose isomerase compared to reported values**

**D**-**xylose isomerase**	***K***_***m ***_**(mM)**	***V***_***max***_^**b**^	**Reference**
*P*. *ruminicola* TC2-24	40	0.28^c^	This work
*P*. *ruminicola* TC2-24^a^	34	0.81^c^	This work
*Piromyces* sp. E2	51	0.25	This work
*Piromyces* sp. E2	20	NA^d^	[12]
*Piromyces* sp. E2	87	0.05	[13]
*Piromyces* sp. E2	50	0.05	[14]
*C*. *phytofermentans*	62	0.03	[14]
*R*. *flavefaciens*	117	NA	[16]
*R*. *flavefaciens*^e^	67	NA	[16]

^a^ After strain adaption.

^b^ μmole/min/mg protein.

^c^ Statistically significant difference (*p* = 0.002).

^d^ Not Available.

^e^ Modified XI.

The measured *V*_*max*_ for the unadapted strain was 0.28 μmole/min/mg protein (Table 3) and was not significantly different from *Piromyces* sp. E2 XI, which was measured at 0.25 μmole/min/mg protein when expressed in our strain. Prior studies have reported *V*_*max*_ for the *Piromyces* XI at 0.05 μmole/min/mg protein (Table 3). Higher enzyme activity than 0.05 μmole/min/mg for the *Piromyces* sp E2 XI has been observed, but *V*_*max*_ was not reported in these studies [12,25,26]. Following strain adaptation, *V*_*max*_ for the *P*. *ruminicola* XI increased 2.9-fold from 0.28 to 0.81 μmole/min/mg protein. This increase probably reflects a higher XI protein concentration in the cell lysate, either from increased expression or stability.

The increase in XI activity for the adapted strain suggested that the copy number of the XI gene may have increased leading to higher protein expression. Gene amplification is a common mechanism for adaption in *S*. *cerevisiae*[33,34] and has been shown to occur under a wide range of circumstances including evolutionary engineering for increased D-xylose metabolism. For instance, one study reported an increase in XI copy number due to integration of up to 32 copies of the *Piromyces* sp. E2 XI gene [6]. To determine if the *P*. *ruminicola* XI gene had integrated into the genome in our adapted strain, we allowed the strain to lose the plasmid containing the XI gene. The strain lacking the XI plasmid was no longer able to grow on D-xylose medium (data not shown), suggesting that integration of the XI gene into the genome did not occur, at least not at levels high enough to support growth on D-xylose in the absence of the XI expression vector. The plasmid containing the XI gene was also rescued from the adapted strain and sequenced. No mutation was found in the XI open reading frame or the promoter or terminator for the gene, indicating that the causative mutation for increased ability to ferment D-xylose resides in the genome. Consistent with this hypothesis, replacing the XI vector in the adapted strain with the original parent XI vector restored the increased growth rate on D-xylose, similar to the original adapted strain (data not shown) while putting the same vector into the unadapted CEN.PK2-1C parent did not. Further analysis of the genome sequence of the adapted strain compared to the parent should reveal the genomic changes responsible for increased D-xylose fermentation.

## Conclusions

Three D-xylose isomerases originating from rumen and gastrointestinal tract bacteria were successfully expressed in a *S*. *cerevisiae* strain, as judged by growth in aerobic D-xylose cultures. The most promising strain, expressing the XI originating from the rumen bacterium *P*. *ruminicola* TC2-24, was adapted in serial batch cultures. The evolved yeast strain (YRH1114) had a specific growth rate of 0.23 h^-1^ on D-xylose in aerobic culture. In fermentative cultures, it produced 13.6 g/L ethanol in 91 h (0.041 g/g_CDW_/h) with a metabolic yield of 83%. The *P*. *ruminicola* XI had a *V*_*max*_ of 0.81 μmole/min/mg protein and a *K*_*m*_ of 34 mM for D-xylose.

## Methods

### Strains, media, and general methods

*Escherichia coli* strains DH10B, TOP10 (Invitrogen; Carlsbad, CA, USA), NEB5a, and NEB10b (New England Biolabs (NEB); Ipswich, MA, USA) were used for routine maintenance and preparation of plasmids and were grown in LB medium [35]. Microorganisms and plasmids used in this study are listed in Table 4 and Table 5. DNA was transformed into yeast cells using a standard lithium acetate method [36]. Synthetic complete (SC) medium consisted of 6.7 g/L Difco yeast nitrogen base (YNB) (United States Biological; Marblehead, MA, USA), and was supplemented with amino acids as necessary [37]. SC medium was filter sterilized. YP medium (10 g/L yeast extract, 20 g/L bacto-peptone) was autoclaved without carbohydrate. Sterile D-glucose or D-xylose was added separately.

**Table 4:** Microorganisms used in this study

**Strain**	**Genotype (description)**	**Reference**
V601	*Bacteroides uniformis*	This work
V923	*Bacteroides distasonis*	This work
V975	*Bacteroides ovatus*	This work
TC2-24	*Prevotella ruminicola* (NRRL # B-50773)	This work
TC27	*Prevotella ruminicola*	This work
D31d	*Prevotella ruminicola*	This work
20-63	*Prevotella ruminicola*	This work
20-78	*Prevotella ruminicola*	This work
E40a	*Prevotella ruminicola*	This work
E42g	*Prevotella ruminicola*	This work
H15a	*Prevotella ruminicola*	This work
H2b	*Prevotella ruminicola*	This work
118b	*Prevotella ruminicola*	This work
20-92A	*Prevotella ruminicola*	This work
CEN.PK2-1C	*S*. *cerevisiae MAT*a *ura3*-*52 trp1*-*289 leu2*-*3*,*112 his3*Δ*1 MAL2*-*8*^*C*^*SUC2*	Euroscarf
YRH400	D5A + integrated (KanMX4; P_*PGK1*_-*XYL1*-T_*PGK1*_; P_*ADH1*_-*XYL2*-T_*ADH1*_; P_*HXT7*_-*XKS1*-T_*HXT7*_)	[27]
YRH561	CEN.PK2-1C [pRS414, pRS416] (control strain with empty vectors)	This work
YRH562	CEN.PK2-1C [pRH195, pRH218] (low copy *XKS1*, high copy *Piromyces* XI)	This work
YRH563	CEN.PK2-1C [pRH195, pRH351] (low copy *XKS1*, high copy *B*. *uniformis* XI)	This work
YRH564	CEN.PK2-1C [pRH195, pRH352] (low copy *XKS1*, high copy *B*. *distasonis* XI)	This work
YRH565	CEN.PK2-1C [pRH195, pRH353] (low copy *XKS1*, high copy *B*. *ovatus* XI)	This work
YRH587	CEN.PK2-1C [pRH195, pRH367] (low copy *XKS1*, high copy *P*. *ruminicola* XI)	This work
YRH592	CEN.PK2-1C [pRH351, pRH369] (low copy *B*. *uniformis* XK, high copy *B*. *uniformis* XI)	This work
YRH628	CEN.PK2-1C [pRH384] (no *XKS1*, high copy *P*. *ruminicola* XI^a^)	This work
YRH629	CEN.PK2-1C [pRH195, pRH384] (low copy *XKS1*, high copy *P*. *ruminicola* XI^a^)	This work
YRH630	CEN.PK2-1C [pRH385, pRH218] (low copy *P*. *ruminicola* XK^a^, high copy *Piromyces* XI)	This work
YRH631	CEN.PK2-1C [pRH385, pRH384] (low copy *P*. *ruminicola* XK^a^, high copy *P*. *ruminicola* XI^a^)	This work
YRH1114	YRH631 adapted for improved D-xylose fermentation	This work
YRH1136	YRH1114 with XI and XK vectors (pRH384 and pRH385) evicted	This work
YRH1137	YRH114 with XI vector (pRH384) evicted, maintains the *P*. *ruminicola* XK^a^ [pRH385]	This work
YRH1138	YRH114 with XK vector (pRH385) evicted, maintains the *P*. *ruminicola* XI^a^ [pRH384]	This work

^a^ codon adapted for expression in *S*. *cerevisiae.*

**Table 5:** Plasmids used in this study

**Plasmid**	**Description**	**Reference**
pRS414	pBluescript II SK+, *TRP1*, *CEN6*, *ARSH4*	[39]
pRS416	pBluescript II SK+, *URA3*, *CEN6*, *ARSH4*	[39]
pRS426	pBluescript II SK+, *URA3*, 2μ origin	[39]
pJ201	Gene synthesis vector	(DNA2.0)
pRH164	pRS414 + P_*HXT7*_-MCS-T_*HXT7*_	[27]
pRH167	pRS426 + P_*HXT7*_-MCS-T_*HXT7*_	[27]
pRH195	pRS414 + P_*HXT7*_-*S*. *cerevisiae XKS1*-T_*HXT7*_	[27]
pRH218	pRS426 + P_*HXT7*_-*Piromyces* sp. E2 XI-T_*HXT7*_	[40]
pRH325	pCR2.1 TOPO + *Bacteroides uniformis* XI	This work
pRH326	pCR2.1 TOPO + *Bacteroides distasonis* XI	This work
pRH327	pCR2.1 TOPO + *Bacteroides ovatus* XI	This work
pRH351	pRS426 + P_*HXT7*_-*Bacteroides uniformis* XI-T_*HXT7*_	This work
pRH352	pRS426 + P_*HXT7*_-*Bacteroides distasonis* XI-T_*HXT7*_	This work
pRH353	pRS426 + P_*HXT7*_-*Bacteroides ovatus* XI-T_*HXT7*_	This work
pRH357	pCR2.1 TOPO + *Prevotella ruminicola strain* TC2-24 XI	This work
pRH360	pCR2.1 TOPO + *Bacteroides uniformis xylB* (D-xylulokinase)	This work
pRH367	pRS426 + P_*HXT7*_-*Prevotella ruminicola* XI-T_*HXT7*_	This work
pRH369	pRS426 + P_*HXT7*_-*Bacteroides uniformis xylB*-T_*HXT7*_	This work
pRH379	pJ201 + *Prevotella ruminicola* TC2-24 codon-adapted XI	This work
pRH380	pJ201 + *Prevotella ruminicola* 23 codon-adapted XK	This work
pRH384	pRS426 + P_*HXT7*_-*P*. *ruminicola* TC2-24 codon-adapted XI -T_*HXT7*_	This work
pRH385	pRS414 + P_*HXT7*_-*P*. *ruminicola* 23 codon-adapted XK -T_*HXT7*_	This work
pRH544	pRS414 + P_*HXT7*_-*P*. *ruminicola* 23 codon-adapted XK -T_*HXT7*_ rescued from YRH1114	This work
pRH545	pRS426 + P_*HXT7*_-*P ruminicola* TC2-24 codon-adapted XI -T_*HXT7*_ rescued from YRH1114	This work

For all plasmids listed using the *HXT7* promoter, P_*HXT7*_ is the truncated version of the *HXT7* promoter for constitutive expression.

### Cloning of D-xylose isomerase genes for expression in *S*. *cerevisiae*

*Bacteroides* and *Prevotella* strains were obtained from our in house collection (M. Cotta, USDA-ARS, NCAUR, Peoria, IL, USA) and were cultivated anaerobically as described previously [38]. D-xylose isomerase genes from *Bacteroides* spp. were PCR amplified from genomic DNA using primers pairs #312/313, #312/314, and #315/316 for strains V923, V975, and V601 respectively (Table 6). DNA fragments corresponding to the expected size for a D-xylose isomerase gene were removed from the agarose gel, purified, and ligated into pCR2.1 TOPO for sequencing. Each of the DNA fragments isolated from the *Bacteroides* spp. PCR reactions encoded a D-xylose isomerase gene. Each XI gene was cloned into the expression vector pRH167 using *Spe*I and *Sal*I restriction endonuclease sites added to the end of the primer used for amplification.

**Table 6:** DNA oligonucleotides used in this study

**#**	**Sequence**
243	5’-TT**ATG**GCWACAAAAGARTWTTTYCCSGG-3’
244	5’-CCTYAGCARTACATATTYASRATKGC-3’
310	5’-GC*ACTAGT***ATG**GCAAAAGAGTATTTCCC-3’
311	5’-CC*GTCGAC*TTACTTGCAGTAAAGTGCTACG-3’
312	5’-GG*ACTAGT***ATG**GCTACAAAAGAGTTTTTTC-3’
313	5’-GC*GTCGAC*TTAGCAGTACATATTTAGGATGG-3’
314	5’-GC*GTCGAC*TTAGCAGTACATATTTACGATGG-3’
315	5’-GG*ACTAGT***ATG**GCTTCAAAAGAGTATTTTC-3’
316	5’-GC*GTCGAC*TTAGCAATACATATTTAGGATGGC-3’

Restriction endonuclease sites are shown italicized and underlined. Start codons are shown in bold.

Since a gene for D-xylose isomerase had not been found in *P*. *ruminicola*, degenerate primers #243 and #244 were used to amplify an isomerase gene from *P*. *ruminicola* strains. Primer #243 was 32-fold degenerate and #244 was 64-fold degenerate. These primers were designed based on *Bacteroides* spp., and *Piromyces* sp. E2. Using these primers and a touch-down PCR cycle, we were unable to successfully amplify a D-xylose isomerase gene from any of the *P*. *ruminicola* strains tested. A DNA fragment containing a D-xylose isomerase gene was isolated when primers based on the XI from sequence from *Prevotella bergensis* (accession # ACKS01000045.1) were used (#310 and #311). The only *P*. *ruminicola* strain to yield a D-xylose isomerase gene by PCR amplification was strain TC2-24. The *P*. *ruminicola* gene from TC2-24 (accession # KC847096) was cloned into pRH167 to create pRH367 for expression of the unoptimized gene in *S*. *cerevisiae*.

Codon-optimized genes were obtained from DNA2.0 (Menlo Park, CA,USA) and cloned into vectors for expression in *S*. *cerevisiae*. The optimized *P*. *ruminicola* (TC2-24) D-xylose isomerase gene was cloned into the high-copy vector pRH167 using the restriction endonucleases *Spe*I and *Sal*I, creating pRH384. To create pRH385 the optimized *P*. *ruminicola* (T23) D-xylulokinase was cloned into the low-copy vector pRH164 using the same restriction endonucleases. Each vector contained the truncated *HXT7* promoter for constitutive expression of the heterologous gene.

### Aerobic growth kinetics

Yeast pre-cultures were grown to mid-log phase in synthetic complete medium with 20 g/L D-glucose and washed with sterile water prior to inoculation. YP medium supplemented with 50 g/L D-xylose (YP5X) was used to determine each yeast strain’s ability to assimilate D-xylose aerobically. Cultures were started using 6-edged flower shaped microtiter plates in 800 μl of YP5X at an OD_660_ of 0.05 and incubated at 30°C, mixing at 1000 rpm using a BioLector® (m2p-labs; Baesweiler, Germany). The BioLector® measures cell density by scattered light, which was measured every 30 minutes. A gain of 20 was used for the experiments to avoid saturation at high optical densities. At this gain, scattered light units were linearly proportional to OD_660_. Scattered light units were converted to optical density using the conversion factor (OD_660_ = background subtracted scattered light unit/22.93). Data shown represent the mean values from experiments that were repeated in triplicate. Standard deviation values were less than 10% of the mean. Specific growth rates for each replicate were determined by linear regression of the natural log transformed data (i.e. background subtracted scattered light units) vs. time. The slope of the fitted line during exponential growth was used as the specific growth rate μ (h^-1^).

Cell culture using the Bioscreen C™ automated microbiology growth curve analysis system (Growth Curves USA; Piscataway, NJ, USA) was performed using 100 well honeycomb plates. Microtiter plates for the Bioscreen C™ have a round geometry unlike the flower-shaped plates used in the BioLector®. Plates were shaken with a high amplitude setting and normal speed setting for 30 seconds with an interval of 60 seconds. OD_660_ was determined by converting the wideband OD values from the Bioscreen C™ using the equation (OD_660_ = 1.74OD + 0.535(OD)^2^ - 0.749(OD)^3^ + 0.675(OD)^4^, R^2^ = 0.999). Data shown represent the mean values from triplicate experiments.

### Strain adaptation

Strain adaptation to aerobic conditions was achieved by serial passage of cells in YP medium supplemented with 50 g/L of D-xylose as the only energy source. Serial passage under aerobic conditions was continued for eight weeks. Strain adaptation for D-xylose fermentation was then performed in duplicate by weekly transfer of cells grown under fermentative conditions (microaerobic) for six weeks. During adaptation to fermentative conditions cultures were sampled at each passage and analyzed for optical density, residual D-xylose, and fermentation products (ethanol, glycerol, xylitol, and acetate).

### Enzyme assays

Total protein from clarified cell lysates was prepared from mid-log phase cells grown in synthetic complete medium using D-glucose as the carbon source. Cells were collected by centrifugation, washed once with sterile ice-cold water, and centrifuged again. Cell pellets were stored at −80°C for later use. Cells were resuspended in an appropriate amount of Y-PER reagent (Pierce; Rockford, IL, USA) plus protease inhibitors (Complete, mini, EDTA-free protease inhibitor cocktail, Roche; Indianapolis, IN, USA) and processed according to the manufacturer’s instructions. Protein concentrations were determined with the Quick Start Bradford Protein Assay (Bio-Rad; Hercules, CA, USA) against a bovine serum albumin standard.

D-xylose isomerase activity was assayed essentially as described in [12]. XI assays were performed using buffer containing 100 mM Tris-HCl, pH 7.5, 10 mM MgCl_2_, 0.15 mM NADH, 2 U sorbitol dehydrogenase (Roche, Mannheim, Germany), and an appropriate amount of cell lysate. Reactions were started by the addition of D-xylose (Sigma; St. Louis, MO, USA) to a final concentration of 500 mM and reactions monitored at 340 nm using a Cary 50 Bio UV-Visible spectrophotometer (Varian; Palo Alto, CA, USA). Specific activity (μmole/min/mg lysate) was determined using the molar absorption coefficient, ϵ_340_, of 6.22 mM^-1^ cm^-1^ for NADH. To ensure that the sorbitol dehydrogenase coupling enzyme was not saturated, the ratio of sorbitol dehydrogenase activity to D-xylose isomerase activity was maintained at > 20 for the assay. Additionally, specific activities reported were proportional to the amount of lysate added in a dilution series. Kinetic parameters were determined by varying the D-xylose concentration from 5 to 500 mM. Each assay was done using lysates prepared from three independent cultures.

### Batch fermentation

D-xylose fermentation was investigated by inoculating 100-ml YP cultures with 50 g/L D-xylose at a starting OD_660_ of 2.0. CO_2_ production was monitored continuously over the course of the fermentation using a wireless gas production measurement system (Ankom Technologies; Macedon, NY, USA). The wireless system monitors gas production indirectly by measuring cumulative gas pressure; CO_2_ production is calculated using the ideal gas law. The system was set to vent when the overhead pressure achieved 1 psi and to monitor the accumulated pressure every 15 min. Exponentially growing cells were used for the inoculum. Post fermentation samples were used to determine cell biomass (by OD_660_), residual sugars, and fermentation products (by high-performance liquid chromatography, HPLC). All fermentation experiments were performed three to four times. Accumulative pressure values were used to calculate the amount of CO_2_ produced during the fermentation. Carbon recoveries were determined using HPLC data. Carbon recovery calculations were based on total input carbon from D-xylose and measured (HPLC) remaining D-xylose, along with fermentation products. CO_2_ amounts used for carbon recovery calculations assumed 1 mole of CO_2_ is produced for every 1 mole of ethanol. Specific ethanol productivity (g ethanol/g cell dry weight/h) was determined using the final OD_660_ measurement (91 hours) for each batch fermentation. Cell dry weight (CDW) was calculated using an OD-to-CDW conversion factor for the yeast strain CEN.PK2-1C (CDW = 0.58 g/L/OD). The conversion factor was determined by drying cells at differing OD to constant weight at 100°C. Cells were washed three times with distilled water prior to drying to remove trace amounts of medium. OD was measured using a GeneSys 10 vis spectrophotomerer (Thermo Fisher Scientific Inc.; Waltham, MA, USA).

### Analytical methods

Extracellular metabolites were measured using HPLC as previously described [27]. Samples were analyzed using a SpectraSYSTEM liquid chromatography system (Thermo Electron Corporation, CA, USA) equipped with an automatic sampler, column heater, isocratic pump, refractive index detector, and computer based integrator running Chromquest ver. 2.5 software (Thermo Electron Corporation). Samples were injected (20 μl) onto a sugar column (Aminex HPX-87H Column, 300 x 7.8 mm, Bio Rad Laboratories, Inc.) and eluted with 5 mM sulfuric acid at 0.6 ml/min and 65°C.

### Statistical analyses

For experiments with three or greater biological replicates, probability analyses were performed using the Student’s *t*-test with a two-tailed distribution and compared to the appropriate control strain. Values with *p* < 0.05 were considered significant for this study. Statistical analysis was performed using Microsoft Excel.

## Abbreviations

XI: D-xylose isomerase; XK: D-xylulokinase; XR: D-xylose reductase; XD: xylitol dehydrogenase; OD: optical density; HPLC: High performance liquid chromatography

## Competing interests

REH, BSD, and MAC are listed on a national pending patent (application number US 13/616,629) for the sequence of the *Prevotella ruminicola* TC2-24 D-xylose isomerase gene and expression of the XI and *P*. *ruminicola* XK for the purpose of D-xylose fermentation.

## Authors’ contributions

REH and BSD performed the work presented in this study. REH designed the study and drafted the manuscript. BSD and JAM participated in design of the study and helped draft the manuscript. MAC supervised the work, and assisted in drafting the manuscript. All authors read and approved the final manuscript.

## Disclaimer

†Mention of trade names or commercial products in this article is solely for the purpose of providing scientific information and does not imply recommendation or endorsement by the US Department of Agriculture. USDA is an equal opportunity provider and employer.

§This article is a US Government work and is in the public domain in the USA.
